# Mortality trends from colorectal pathologies in the United States (1999–2020): a retrospective cohort study using CDC WONDER

**DOI:** 10.1007/s10151-025-03247-8

**Published:** 2026-03-28

**Authors:** J. L. Rogers, D. Ali, A. Khan

**Affiliations:** 1 Vanderbilt University School of Medicine, Nashville, TN USA; 2 Department of Surgery, Section of Surgical Sciences, Vanderbilt University Medical Center, 1161 21st Ave S., Room D5203 MCN, Nashville, TN 372323 USA

**Keywords:** Benign colorectal disease, CDC WONDER, *Clostridioides difficile*, Colorectal cancer, Colorectal mortality, Inflammatory bowel disease

## Abstract

**Background:**

Colorectal diseases encompass both malignant and benign conditions with significant public health implications. While colorectal cancer (CRC) mortality has been well characterized, population-level mortality trends for benign colorectal conditions remain poorly defined.

**Methods:**

This retrospective cohort study analyzed mortality data for U.S. decedents aged ≥15 years from 1999–2020 using the CDC WONDER database. Underlying causes of death were categorized as CRC, inflammatory bowel disease (IBD), or benign colorectal conditions. Crude death rates (CDRs) per million were calculated, and temporal trends were evaluated using Joinpoint regression to estimate annual percent change (APC).

**Results:**

Of 56,014,102 total deaths, 1,560,448 (2.7%) were due to colorectal pathologies. CRC accounted for most deaths (CDR 216.2), followed by benign colorectal conditions (68.5) and IBD (3.8) (p0.001). Among benign causes, Clostridioides difficile enterocolitis (CDR 21.6) was the leading contributor, followed by diverticular disease (12.4) and acute vascular intestinal disorders (9.0). CRC mortality declined significantly (AAPC –1.44%, p0.05), whereas benign colorectal and IBD mortality remained stable.

**Conclusions:**

Mortality from benign colorectal conditions has persisted over two decades despite major declines in CRC mortality, highlighting unmet needs in research and prevention. Future public health efforts should extend beyond malignancy to address preventable deaths from benign colorectal disease.

## Introduction

Colorectal pathologies are diverse, ranging from malignancies to benign disorders. Among them, colorectal cancer (CRC), which remains a leading cause of cancer-related mortality worldwide, has garnered significant clinical and research attention [1, 2]. Compared to CRC, benign colorectal conditions are comparatively understudied at a population level, despite their clinical relevance. Many such conditions are medically managed, falling outside the scope of widely used national surgical databases [3, 4]. Accordingly, mortality trends in benign colorectal conditions are poorly characterized, leaving a gap regarding their public health impact.

The study objectives were to (1) assess trends in mortality from benign colorectal conditions in the USA over the past two decades and compare them to CRC and inflammatory bowel disease (IBD) and (2) identify leading causes of mortality from benign colorectal conditions. In addition to CRC, we included IBD to represent chronic inflammatory conditions with established links to CRC risk and significant long-term morbidity. Together with benign colorectal conditions, these groups reflect the spectrum of colorectal pathologies that contribute to mortality and public health burden.

## Methods

This retrospective study, conducted in accordance with STROBE guidelines, utilized publicly available data from the Centers for Disease Control and Prevention Wide-Ranging Online Data for Epidemiologic Research (CDC WONDER) database (https://wonder.cdc.gov/), which captures all deaths occurring in the USA. The study cohort included all US decedents aged ≥ 15 years with a colorectal pathology as an underlying cause of death from 1999 to 2020. The underlying cause of death was categorized as CRC, IBD, or benign, which included all non-IBD-related benign colorectal conditions (Table 1). The underlying cause of death is based on the World Health Organization criteria as the disease that directly led to the sequence of events leading to the death of the individual. To compare mortality among benign colorectal conditions, CRC, and IBD, we calculated annual crude death rates (CDRs) using the following formula:

$${\text{CDR = }}\frac{{\text{Deaths from specific disease }}}{{{\text{US population }}\left( {\text{US Census Bureau estimates}} \right)}}\user2{ }\times\user2{ }1,000,000$$

**Table 1:** Total deaths and CDR by colorectal disease subtype (cancer, IBD, and benign conditions) among patients 15–85+ years of age

	Disease (ICD-10 code)	Total population (*A*), *N* = 5,409,649,211		*p* value
		Deaths (*B*) 56,014,102	CDR^a^	
	Benign	370,795 (0.662%)	68.54	
	Cancer	1,169,336 (2.088%)	216.16	< 0.001*
	IBD	20,317 (0.036%)	3.76	< 0.001*
Cancer	Malignant neoplasm of colon (C18)	957,182 (1.709%)	176.94	
	Malignant neoplasm of rectum (C20)	148,862 (0.266%)	27.52	
	Malignant neoplasm of rectosigmoid junction (C19)	60,055 (0.107%)	11.1	
	Neoplasm of uncertain behavior of colon (D37.4)	2564 (0.005%)	0.47	
	Neoplasm of uncertain behavior of rectum (D37.5)	673 (0.001%)	0.12	
IBD	Crohn disease (K50)	13,119 (0.023%)	2.43	
	Ulcerative colitis (K51)	7198 (0.013%)	1.33	
Benign	Enterocolitis due to *Clostridium difficile* (A04.7)	116,979 (0.209%)	21.62	
	Diverticular disease of intestine (K57)	67,310 (0.120%)	12.44	
	Acute vascular disorders of intestine (K55.0)	48,560 (0.007%)	8.98	
	Perforation of intestine (nontraumatic) (K63.1)	45,502 (0.081%)	8.41	
	Diarrhea and gastroenteritis of infectious origin (A09)	31,851 (0.057%)	5.89	
	Noninfective gastroenteritis and colitis, unspecified (K52.9)	21,537 (0.038%)	3.98	
	Volvulus (K56.2)	13,314 (0.024%)	2.46	
	Megacolon, not elsewhere classified (K59.3)	4796 (0.009%)	0.89	
	Chronic vascular disorders of intestine (K55.1)	4574 (0.008%)	0.85	
	Fistula of intestine (K63.2)	4161 (0.007%)	0.77	
	Constipation (K59.0)	3363 (0.006%)	0.62	
	Hemorrhage of anus and rectum (K62.5)	3131 (0.006%)	0.58	
	Other specified bacterial intestinal infections (A04.8)	1481 (0.003%)	0.27	
	Benign neoplasm of colon, rectum, anus and anal canal (D12)	835 (0.001%)	0.15	
	Ulcer of intestine (K63.3)	749 (0.001%)	0.14	
	Benign neoplasm of colon, unspecified (D12.6)	689 (0.001%)	0.13	
	Rectal prolapse (K62.3)	618 (0.001%)	0.11	
	Polyp of colon (K63.5)	547 (0.001%)	0.1	
	Irritable bowel syndrome (K58)	454 (0.001%)	0.08	
	Toxic gastroenteritis and colitis (K52.1)	198 (< 0.001%)	0.04	
	Other specified noninfective gastroenteritis and colitis (K52.8)	134 (< 0.001%)	0.02	
	Other intestinal *Escherichia coli* infections (A04.0)	12 (< 0.001%)	0	

Total population and total deaths were calculated from 1999 to 2020

*CDR* crude death rate, *IBD* inflammatory bowel disease

*Statistically significant

^a^CDR was calculated using the following formula: (*B*/*A*) × 1,000,000. Therefore, CDR is reported per one million people

Differences in mortality distributions among groups were evaluated using chi-square tests in Microsoft Excel, v16.96.1 (Microsoft Corporation, Armonk, NY). Joinpoint v5.4.0 enabled quantification of mortality trends using annual percent change (APC). Statistical significance was set at *p* < 0.05.

## Results

**Fig. 1:**
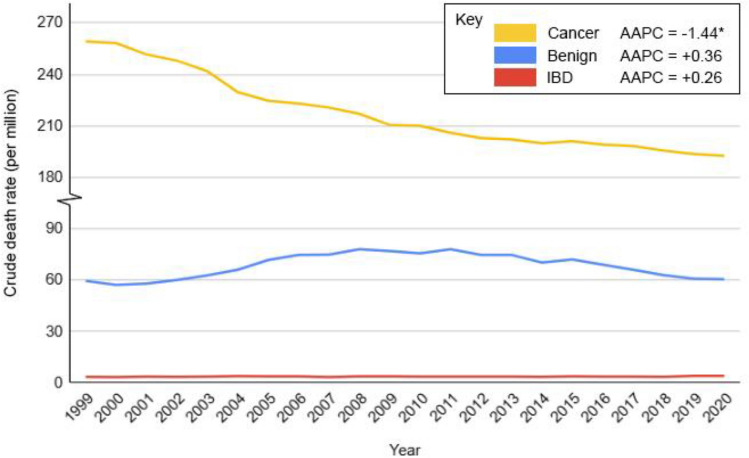
Temporal trends in crude death rates for colorectal cancer, benign colorectal conditions, and inflammatory bowel disease (IBD) in the USA, 1999–2020. APC for colorectal cancer: 1999–2009 = − 2.20*, 2009–2020 = − 0.78*; APC for benign disorders: 1999–2009 =  + 3.70*, 2009–2020 = − 2.47*; APC for IBD: 1999–2018 =  + 0.05, 2018–2020 =  + 6.34*. *Indicates that the AAPC or APC is significantly different (*p* < 0.05) from zero. *Cancer* colorectal cancer, *IBD* inflammatory bowel disease, *AAPC* average annual percentage change, *APC* annual percentage change

**Fig. 2:**
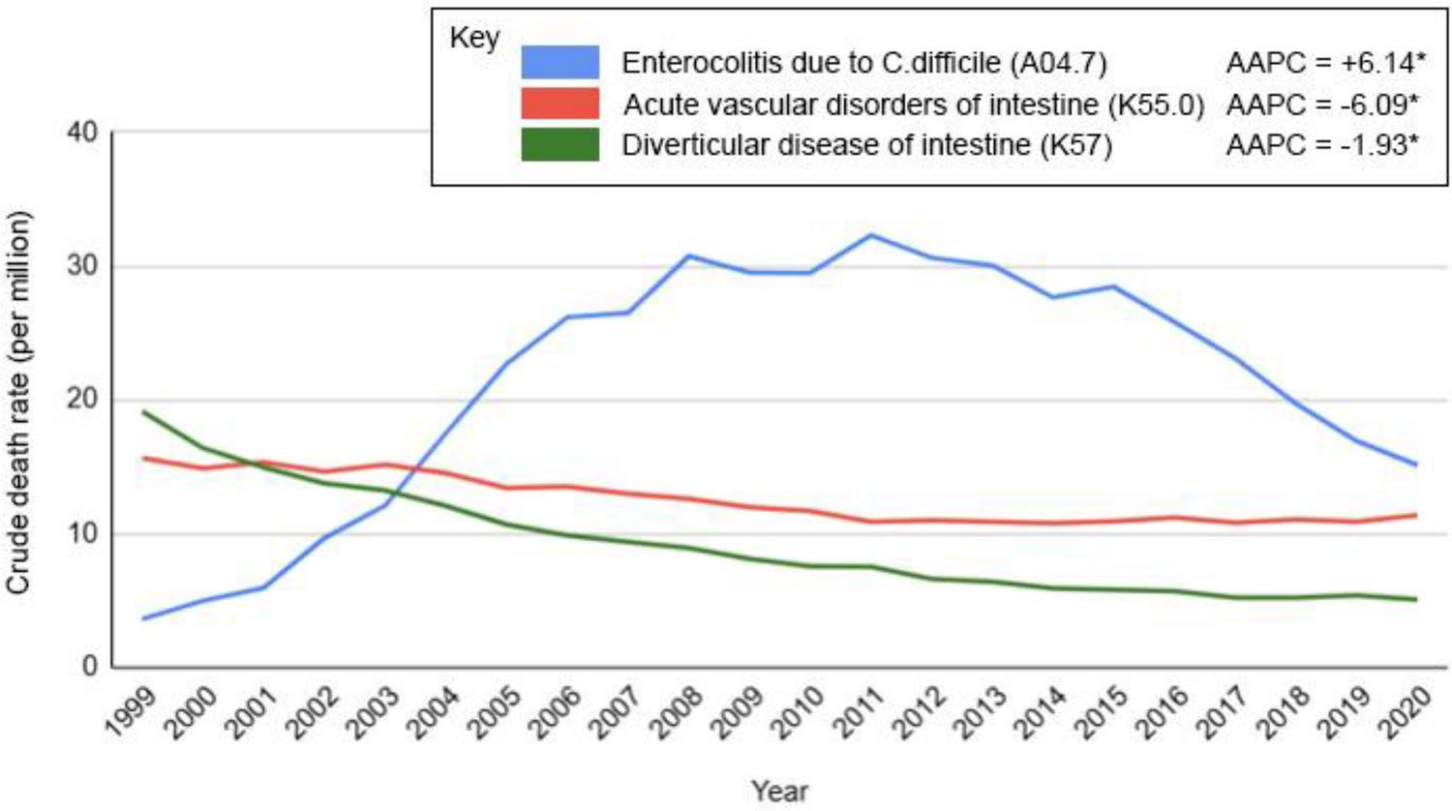
Temporal trends in crude death rates for the three most common benign colorectal conditions in the USA, 1999–2020. APC for enterocolitis due to *C. difficile*: 1999–2006 =  + 34.55*, 2006–2015 =  + 0.16, 2015–2020 = − 12.69*; APC for acute vascular disorders of intestine: 1999–2012 = − 7.48*, 2012–2020 = − 3.22*; APC for diverticular disease of intestine: 1999–2013 = − 2.82*, 2013–2020 = + 0.53. ICD-10 codes for the three most common pathologies are in parentheses. *Indicates that the AAPC or APC is significantly different (*p* < 0.05) from zero. *C. difficile Clostridium difficile*, *AAPC* average annual percentage change, *APC* annual percentage change

Of the 56,014,102 deaths recorded in the USA over the study period, 1,560,448 (2.7%) were attributed to colorectal pathologies. CRC accounted for the majority of deaths, significantly exceeding deaths from benign colorectal conditions (CDR 216.16 vs. 68.54, *p* < 0.001). Benign colorectal condition mortality was higher than that from IBD throughout the study period (CDR 68.54 vs. 3.76, *p* < 0.001). Overall, the leading causes of death were malignant neoplasms of the colon (CDR 176.94) followed by malignant neoplasms of the rectum (CDR 27.5). Among benign pathologies, *Clostridioides difficile* enterocolitis (CDR 21.62) was the most common cause of death, followed by diverticular disease of the intestine (CDR 12.44) and acute vascular intestinal disorders (CDR 8.98) (Table 1). IBD CDR was 3.76. Temporal trends revealed that CRC-related mortality declined over time (AAPC = − 1.44%, *p* < 0.05), whereas mortality from benign colorectal conditions (AAPC = 0.36%, *p* > 0.05) and IBD (AAPC = 0.26%, *p* > 0.05) remained stable (Figs. 1 and 2).

## Discussion

Benign colorectal conditions account for a sustained and substantial proportion of mortality among colorectal pathologies. Unlike CRC, which shows a decreasing trend [1], mortality from benign conditions and IBD have remained the same over the past two decades with a slow sub percentage point annual growth. The decline in CRC mortality is likely multifactorial, but coincides with decades of targeted investment in screening programs, public awareness efforts, and therapeutic innovation, largely supported by the National Cancer Institute. Conversely, benign colorectal conditions and IBD have not received equivalent research or policy attention [2]. Despite their comparatively lower death rates, these conditions continue to account for a substantial number of deaths annually.

This is the first study highlighting national trends in benign colorectal condition mortality. While the increase in *C. difficile* enterocolitis mortality has not been evaluated on a national level, increasing mortality rates beyond 2010 align with past evidence of its prevalence in hospital settings and hypervirulent strains [3, 4]. Despite increasing focus on antimicrobial stewardship, population-level mortality improvements have plateaued. Diverticular disease similarly remains a major contributor to colorectal death. Recent evidence suggests rising prevalence [5, 6]; however, there is a paucity of data on mortality. A decrease in mortality either suggests a shift towards less severe disease pathology or earlier detection and more aggressive management [7]. Mortality from acute vascular disorders of the intestine has also trended downward, possibly due to improved mesenteric ischemia imaging, earlier risk factor recognition, and increased vascular disease screening [8].

Limitations of this study include reliance on death certificate data, which can be affected by diagnostic misclassification and does not capture disease severity or treatment history. However, the large, nationally representative dataset used over a 21-year span strengthens the validity of observed trends. Another limitation of this study is the reliance on crude death rates, which may be influenced by changes in population age structure over time. Age-adjusted rates could provide complementary insights but were beyond the scope of this analysis. Lastly, benign colorectal conditions are less systematically tracked in national registries, which may contribute to underdiagnosis or underreporting and influence observed mortality trends.

In conclusion, CRC mortality has declined substantially, likely due to long-term national investment in screening and therapeutic innovation. However, mortality from benign colorectal conditions and IBD has remained stable [9, 10], reflecting ongoing public health gaps. The decline in deaths from acute vascular intestinal disorders is encouraging, potentially owing to improved diagnostics and individualized care. Future efforts should extend the progress observed in CRC outcomes to benign colorectal conditions through increased research investment.

## Data Availability

No datasets were generated or analysed during the current study.

## References

[1] Tan JY, Yeo YH, Ng WL, Fong ZV, Brady JT (2024) How have US colorectal cancer mortality trends changed in the past 20 years? Int J Cancer 155(3):493–500 38525799 10.1002/ijc.34926

[2] Ballreich JM, Gross CP, Powe NR, Anderson GF (2021) Allocation of National Institutes of Health funding by disease category in 2008 and 2019. JAMA Netw Open 4(1):e2034890–e2034890 33502486 10.1001/jamanetworkopen.2020.34890 PMC7841468

[3] Guh AY, Mu Y, Winston LG et al (2020) Trends in US burden of *Clostridioides difficile* infection and outcomes. N Engl J Med 382(14):1320–1330 32242357 10.1056/NEJMoa1910215 PMC7861882

[4] Hall AJ, Curns AT, McDonald LC, Parashar UD, Lopman BA (2012) The roles of *Clostridium difficile* and norovirus among gastroenteritis-associated deaths in the United States, 1999–2007. Clin Infect Dis 55(2):216–223 22491338 10.1093/cid/cis386

[5] Khan A, Hawkins AT (2021) Challenging surgical dogma: controversies in diverticulitis. Surg Clin North Am 101(6):967–980 34774275 10.1016/j.suc.2021.05.024 PMC8593444

[6] Wheat CL, Strate LL (2016) Trends in hospitalization for diverticulitis and diverticular bleeding in the United States from 2000 to 2010. Clin Gastroenterol Hepatol 14(1):96–103 25862988 10.1016/j.cgh.2015.03.030 PMC4624035

[7] Hawkins AT, Wise PE, Chan T et al (2020) Diverticulitis: an update from the age old paradigm. Curr Probl Surg 57(10):100862 33077029 10.1016/j.cpsurg.2020.100862 PMC7575828

[8] Bharucha AE, Parthasarathy G, Ditah I et al (2015) Temporal trends in the incidence and natural history of diverticulitis: a population-based study. Am J Gastroenterol 110(11):1589–1596 26416187 10.1038/ajg.2015.302 PMC4676761

[9] Javaid SS, Akhtar S, Hafeez A et al (2025) Trends in mortality due to inflammatory bowel disease in the United States: a CDC WONDER database analysis (1999–2020). Dig Dis Sci. 10.1007/s10620-024-08803-0 39746892 10.1007/s10620-024-08803-0

[10] Ye Y, Manne S, Treem WR, Bennett D (2020) Prevalence of inflammatory bowel disease in pediatric and adult populations: recent estimates from large national databases in the United States, 2007–2016. Inflamm Bowel Dis 26(4):619–625 31504515 10.1093/ibd/izz182

